# Approximate confidence intervals for moment‐based estimators of the between‐study variance in random effects meta‐analysis

**DOI:** 10.1002/jrsm.1162

**Published:** 2015-08-19

**Authors:** Dan Jackson, Jack Bowden, Rose Baker

**Affiliations:** ^1^ Biostatistics UnitMRCCambridgeUK; ^2^ School of BusinessUniversity of SalfordSalfordUK

**Keywords:** interval estimation, meta‐regression, quadratic form, large sample inference

## Abstract

Moment‐based estimators of the between‐study variance are very popular when performing random effects meta‐analyses. This type of estimation has many advantages including computational and conceptual simplicity. Furthermore, by using these estimators in large samples, valid meta‐analyses can be performed without the assumption that the treatment effects follow a normal distribution. Recently proposed moment‐based confidence intervals for the between‐study variance are exact under the random effects model but are quite elaborate. Here, we present a much simpler method for calculating approximate confidence intervals of this type. This method uses variance‐stabilising transformations as its basis and can be used for a very wide variety of moment‐based estimators in both the random effects meta‐analysis and meta‐regression models. © 2015 The Authors. Research Synthesis Methods published by John Wiley & Sons, Ltd.

## 1 Introduction

The random effects model for meta‐analysis (Biggerstaff and Tweedie, 1997; DerSimonian and Laird, 1986; Hardy and Thompson, 1996) is now widely used. This model includes a random effect that describes the extent of the between‐study variation. The variance of this random effect is called the ‘between‐study variance’, and here, the focus is on performing interval estimation for this parameter. Although inference for the average treatment effect is of primary interest, accurately quantifying the extent of the between‐study variance is an important secondary consideration.

The most popular estimator of the between‐study variance is the moment‐based estimator proposed by DerSimonian and Laird (1986), which has since been put into more general estimation frameworks (DerSimonian and Kacker, 2007; Rukhin, 2013). One major advantage of these point estimates is that they do not require the normality assumptions made by the random‐effects model. This means that, in large samples (meta‐analyses with sufficient numbers of large studies, so that both the between and the within‐study variances can be well approximated by their estimates; the central limit theorem then ensures the pooled estimate is approximately normally distributed), valid random effects meta‐analyses can be performed without making normality assumptions. By a valid random effects meta‐analysis we mean an analysis where the bias in the pooled estimate is negligible and where the actual coverage probability of the confidence interval for the average treatment effect is very close to the nominal level. The standard confidence interval for the treatment effect will be valid approximately in a distribution‐free context when there are many studies (Higgins *et al*., 2009), but in smaller samples, we require normality assumptions to justify the use of a normal approximation for the pooled estimate.

Confidence intervals based on these moment‐based estimators, which are exact under the random effects model, have recently been developed (Biggerstaff and Jackson, 2008; Jackson, 2013). It is important to recognise that these ‘exact’ methods are only exact under the random effects model. As explained in Section 2, this model makes some strong assumptions, including fixed and known within‐study variances. Some may therefore describe our exact confidence intervals as ‘non‐approximate’ or ‘small sample’ confidence intervals, in order to avoid any connotations of the word ‘exact’. For real datasets, such as those in Section 5, all confidence intervals are only approximate because the random effects model merely provides an approximation in practice. A further difficulty is that these ‘exact’ confidence intervals require the use of iterative methods and algorithms (Farebrother, 1984) and so are computationally and conceptually quite complex. The intention here is to provide a much simpler method for producing approximate confidence intervals of this type. An alternative exact method for obtaining confidence intervals is the *Q* profile method (Viechtbauer, 2007). Efficient methods for obtaining confidence intervals using this method are now available (Jackson *et al.*, 2014), but in contrast to the method proposed here, iterative methods are also needed when using the *Q* profile method.

Moment‐based estimators for the between‐study variance have also been proposed for the random effects meta‐regression model (Knapp and Hartung, 2003; Jackson *et al.*, 2014). In the context of meta‐regression, the between‐study variance represents the variation in the study estimates that is not explained by the covariates. This variance is usually referred to as the *residual* between‐study variance in the context of a meta‐regression model. The exact methods for calculating confidence intervals for the between‐study variance in random‐effects meta‐analysis have recently been extended to the meta‐regression setting (Jackson *et al.*, 2014). The proposed approximate method is derived below in the more general framework of meta‐regression, so that the necessary results for meta‐analysis are recovered as the special case of an intercept only (no covariates) regression. Hence, our methods are applicable to both random‐effects meta‐analysis and meta‐regression models, but the former type of model provides our main interest.

The rest of the paper is set out as follows. In Section 2, we briefly review the random effects model. In Section 3, we present our new approximate method for calculating confidence intervals. In Section 4, we perform a simulation study, and in Section 5, we apply our methods to some real datasets. We conclude with a discussion in Section 6.

## 2 The random effects model

We will present our methods in terms of a general random effects meta‐regression model. The necessary methods for meta‐analysis are obtained as a special case from the results presented in Sections 2 and 3.

The random effects meta‐regression model assumes that $Y_{i}|x_{i}∼N\left(x_{i}\beta ,\sigma_{i}^{2}+\tau^{2}\right)$, where *Y*_*i*_ is the estimated effect from the *i*th study, *i* = 1, 2, …, *n*, **x**_*i*_ is the 1 × *p* row vector of covariates associated with this study and *β* is the vector of regression parameters of interest. For a standard meta‐analysis simply focusing on an overall effect, with no adjustment for covariates, **x**_*i*_ = 1 for all *i*. The parameter *τ* ^2^ is the between‐study (in a meta‐regression, residual) variance. The within‐study variances $\sigma_{i}^{2}$ are estimated in practice but are treated as fixed and known in analysis. Our aim is to provide a simple approximate method for calculating confidence intervals for *τ* ^2^.

It is important to recognise that the random‐effects model makes a number of assumptions and can be quite a crude approximation when applied to real data. These assumptions include within and between‐study normality where the within‐study variances are replaced by their estimates. These assumptions greatly simplify the mathematics. All the confidence intervals for *τ* ^2^ calculated in this paper, *unlike* the point estimates, require the normality assumptions made by the random effects model. Our position is that the methods that follow can be used in situations where the random‐effects model is considered to be a suitable approximation. In current practice, the random‐effects model is generally considered to be widely applicable and is routinely used. The random effects model provides a reasonable approximation in situations where the studies are large enough to justify the use of normal approximations within‐studies and the assumption of between‐study normality is considered to be reasonable. However, Kulinskaya *et al*. (2011a, 2011b) show that results concerning quadratic forms in meta‐analysis that rely on the assumptions of the random effects model can be poor approximations when applied to real datasets, and all the methods that follow are subject to these issues. Methods that take into account the fact that the within‐study variances are estimated have been proposed (Böhning *et al.,* 2002; Malzahn *et al.,* 2000), but at present, our methodology does not attempt this. Extending our methods in order to acknowledge the uncertainty in the estimates of the within‐study variances is a very important avenue for further research.

To produce our methodology, we will require frequent use of matrix algebra. The matrix formulation of the random effects meta‐regression model is

(1) $$Y|X∼N\left(X\beta ,\Delta +\tau^{2}I\right)$$

where **Y** is a column vector of length *n* containing the *Y*_*i*_, **X** is the *n* × *p* design matrix (sometimes referred to as the model matrix) whose *i*th row is **x**_*i*_, $\Delta =\text{diag}\left(\sigma_{i}^{2}\right)$ (i.e. Δ is the diagonal matrix containing the $\sigma_{i}^{2}$) and **I** is the *n* × *n* identity matrix. We will also define Σ = Δ + *τ* ^2^ ***I***.

DerSimonian and Kacker (2007) proposed using a generalised version of Cochran's *Q* statistic (Cochran, 1954; Biggerstaff and Jackson, 2008) in the special case of meta‐analysis. This uses an arbitrary set of fixed positive constants *a*_*i*_ instead of $w_{i}=\sigma_{i}^{-2}$ when computing *Q*. As explained by Jackson *et al.* (2014), an obvious and more general version of DerSimonian and Kacker's heterogeneity statistic for meta‐regression is

$$Q_{a}=\sum_{i=1}^{n}\;a_{i}{\left(Y_{i}-{\hat{Y}}_{i}\right)}^{2}$$

where the *a*_*i*_ are arbitrary positive constants and ${\hat{Y}}_{i}=x_{i}{\hat{\beta }}_{a}$, where ${\hat{\beta }}_{a}={\left(X^{t}\text{AX}\right)}^{-1}X^{t}AY$ and **A** = diag(*a*_*i*_). If *a*_*i*_ = *w*_*i*_ for all *i*, then *Q*_*a*_ reduces to the usual *Q* statistic for meta‐regression (Knapp and Hartung, 2003), and if further there are no covariates, then, because we use the approximation that the within‐study variances are fixed and known, *Q*_*a*_ reduces to Cochran's heterogeneity statistic for meta‐analysis. We use the symbol *Q*_*a*_ to emphasise that *Q* depends on the weights *a*_*i*_. The same weights are used for calculating ${\hat{\beta }}_{a}$ and *Q*_*a*_. Hence, the same intuition for choosing weights applies throughout. Intuitively appealing weights include the reciprocal of the within‐study variances or the reciprocal of the standard errors.

In order to derive the properties of *Q*_*a*_, we write this in matrix form. We have that

$$Y-\hat{Y}=\left(I-X{\left(X^{t}\text{AX}\right)}^{-1}X^{t}A\right)Y$$

so that *Q*_*a*_ can be written as

$$Q_{a}={\left(Y-\hat{Y}\right)}^{t}A\left(Y-\hat{Y}\right)$$

and after further manipulation, we can write

$$Q_{a}=Y^{t}BY$$

where **B** = **A** − **AX**(**X** ^*t*^ **AX**)^− 1^ **X** ^*t*^ **A**. In the case of a meta‐analysis (no covariates), the matrix **B** has the particularly simple form $B=A-\frac{1}{a_{+}}aa^{t}$, where **a** is the vector containing the *a*_*i*_ and *a*_+_ = ∑_*i*_ *a*_*i*_. Writing **Y** = **X** *β* + **Z**, because ***B*X** = **0**, we can write

(2) $$Q_{a}=Y^{t}BY=Z^{t}BZ$$

where **Z** ∼ *N*(**0**, Σ). Equation (2) provides the basis of the approximate method for calculating confidence intervals for *τ* ^2^ that follows in Section 3. The exact method for calculating confidence intervals (Biggerstaff and Jackson, 2008; Jackson, 2013; Jackson *et al.*, 2014) is based on writing *Q*_*a*_ as a linear combination of *χ* ^2^ random variables, but here, we instead base our approximate method on the first two moments of *Q*_*a*_ and use a variance‐stabilising transformation.

Such transformations have long been used in statistical work, Fisher's *z* transformation of the correlation coefficient (Fisher, 1915) probably being the earliest example. More recently, Lyles and Kupper (1999) used them to obtain improved confidence intervals, and they are currently widely used in the life sciences.

## 3 Approximate confidence intervals for *τ* ^2^

Kulinskaya *et al.* (2008), chapter 22, present variance stabilisation for the non‐central *χ* ^2^ distribution, but here, we stabilise the variance of *Q*_*a*_. Searle (1971), Theorem 1 on page 55 and Corollary 1.3 on page 57 (with a slight clash of notation to ours), state that when **x** ∼ *N*(**0**, **V**) we have that E(**x** ^*t*^ **Ax**) = tr(**AV**) and Var(**x** ^*t*^ **Ax**) = 2tr(**AVAV**). The first of these results for the expectation of the quadratic form is also true when **x** is non‐normal (Searle, 1971, page 55). Applying these results to *Q*_*a*_ in (2), with the modelling assumptions made in (1), immediately results in

(3) $$E\left(Q_{a}\right)=tr\left(B\Sigma \right)=tr\left(B\Delta \right)+tr\left(B\right)\tau^{2}$$

and

(4) $$Var\left(Q_{a}\right)=2tr\left(B\Sigma B\Sigma \right)=2tr\left(B\Delta B\Delta \right)+4\tau^{2}tr\left(B\Delta B\right)+2\tau^{4}tr\left(B^{2}\right)$$

All matrices on the right‐hand side of (3) and (4) are constants under the random effects model because the weights *a*_*i*_ and the within‐study variances $\sigma_{i}^{2}$ are constants under this model. Using weights that are derived from estimates invalidate the theory, but we continue to use the standard approximation of taking the within‐study variances, so that the weights that follow are regarded as known. Caution is however required when using this approximation when some of the studies are small.

Equation (3) results in a moment‐based estimator for *τ* ^2^ by replacing E(*Q*_*a*_) with the observed value of *Q*_*a*_, and *τ* ^2^ with ${\hat{\tau }}^{2}$, and then solving for ${\hat{\tau }}^{2}$. Because the first of Searle's results above is true without the assumption of normality, (3) provides an estimate of *τ* ^2^ without requiring normality assumptions. The estimate ${\hat{\tau }}^{2}$ is usually truncated when the solution of the estimating equation is negative. In the context of meta‐analysis, upon using *a*_*i*_ = *w*_*i*_, this procedure results in the famous DerSimonian and Laird (1986) estimator.

We will base our approximation on the ‘untruncated’ estimate of *τ* ^2^ resulting from (3), ${\hat{\tau }}_{a}^{2}=\left(Q_{a}-tr\left(B\Delta \right)\right)/tr\left(B\right)$. Then (3) and (4) imply that

(5) $$E\left({\hat{\tau }}_{a}^{2}\right)=\tau^{2}$$

and

(6) $$Var\left({\hat{\tau }}_{a}^{2}\right)=C_{0}+C_{1}\tau^{2}+C_{2}\tau^{4}$$

where *C*_0_ = 2tr(**B**Δ**B**Δ)/(tr(**B**)^2^), *C*_1_ = 4tr(**B**Δ**B**)/(tr(**B**)^2^) and *C*_2_ = 2tr(**B** ^2^)/(tr(**B**)^2^).

### 3.1 The variance stabilising transformation for the ‘untruncated’ estimate of *τ* ^2^

From the moments in (5) and (6), the variance‐stabilising transformation for ${\hat{\tau }}_{a}^{2}$ is

(7) $$f\left(x;C_{0},C_{1},C_{2}\right)=\int^{x}\frac{du}{\sqrt{C_{0}+C_{1}u+C_{2}u^{2}}}=\frac{1}{\sqrt{C_{2}}}\log\left\{2C_{2}x+C_{1}+2\sqrt{C_{2}\left(C_{2}x^{2}+C_{1}x+C_{0}\right)}\right\}$$

The integral in (7) is a standard result when *C*_2_ > 0 (Jeffrey and Zwillinger, 2014), which can be verified by differentiating the integrand in the right‐hand side of (7) and rearranging the resulting expression.

Although it is clear that *g*(*x*) = *C*_0_ + *C*_1_ *x* + *C*_2_ *x* ^2^ > 0 for *x* ≥ 0, because *g*(*x*) is the variance of *Q*_*a*_ when *τ* ^2^ = *x*, to make convenient statistical use of (7), we also require that *g*(*x*) > 0 for − tr(**B**Δ)/tr(**B**) ≤ *x* < 0. This will be true if the discriminant of the quadratic $C_{1}^{2}-4C_{0}C_{2}=-\delta^{2}<0$. In the Appendix, we prove that this condition is satisfied.

With *δ* > 0, we can gain some insight into the nature of the transformation (7) by writing *C*_0_ + *C*_1_ *u* + *C*_2_ *u* ^2^ = *C*_2_{(*u* − *β*)^2^ + *α* ^2^}, where from equating coefficients *β* = − *C*_1_/2*C*_2_, *α* = *δ* ^1/2^/2*C*_2_. Then, we change variable to *u* = *β* + *α* sinh(*y*), when d*u* = *α* cosh(*y*)d*y*, where 2 sinh(*y*) = exp(*x*) − exp(−*x*) and 2 cosh(*y*) = exp(*x*) + exp(−*x*), and the integral is

$$\frac{1}{\sqrt{C_{2}}}\text{arcsinh}\left\{\left(2C_{2}x+C_{1}\right)/\delta^{1/2}\right\}=\frac{1}{\sqrt{C_{2}}}\text{log}\left\{\left(2C_{2}x+C_{1}\right)/\delta^{1/2}+\sqrt{1+{\left(2C_{2}x+C_{1}\right)}^{2}/\delta }\right\}$$

We regain (7) on adding the constant of integration $\text{log}\left(\delta \right)/\left(2\sqrt{C_{2}}\right)$. This shows that the variance‐stabilising transformation is Johnson's arcsinh transformation (Johnson, 1949), which is often used to normalise distributions.

### 3.2 Obtaining confidence intervals for *τ* ^2^

From the linear approximation that underlies the variance stabilising transformation (7), we have the approximation that

$$f\left({\hat{\tau }}_{a}^{2};C_{0},C_{1},C_{2}\right)∼N\left(f\left(\tau^{2};C_{0},C_{1},C_{2}\right),1\right)$$

so that an approximate 100(1 − *α*)% confidence interval for *τ* ^2^ is given by

(8) $$\left[f^{-1}\left(f\left({\hat{\tau }}_{a}^{2};C_{0},C_{1},C_{2}\right)-Z_{\alpha /2};C_{0},C_{1},C_{2}\right),f^{-1}\left(f\left({\hat{\tau }}_{a}^{2};C_{0},C_{1},C_{2}\right)+Z_{\alpha /2};C_{0},C_{1},C_{2}\right)\right]$$

where *Z*_*α*/2_ is the 100(1 − *α*/2)% percentile of a standard normal density and [*a*, *b*] is the interval from *a* to *b*. For example, for a 95% confidence interval, we use *Z*_0.975_ = 1.96. It is also possible to use different probabilities in each tail (with their sum equalling *α*) to define a confidence interval, but here, we follow the common convention of using ‘equal tailed’ confidence intervals.

Although the function *f* is quite complicated, an advantage of the proposed interval is that its inverse function is of a particularly simple form, which follows from the fact that it is an arcsinh. This inverse can be obtained by exploiting that fact or directly from (7): We write *y* = *g*(*x*) and rearrange the resulting equation until we obtain *x* = *h*(*y*) and *g* ^− 1^(⋅) = *h*(⋅). Then, the necessary rearrangement begins by multiplying both sides of *y* = *g*(*x*) by $\sqrt{C_{2}}$, exponentiating and then subtracting (2*C*_2_ *x* + *C*_1_) from both sides, and then squaring both sides. The *x* ^2^ terms cancel, and the resulting inverse function is

$$f^{-1}\left(x;C_{0},C_{1},C_{2}\right)=\frac{\text{exp}\left(\sqrt{C_{2}}x\right)-2C_{1}+\left(C_{1}^{2}-4C_{0}C_{2}\right)\text{exp}\left(-\sqrt{C_{2}}x\right)}{4C_{2}}$$

This inverse function can be used to provide the two confidence interval bounds in (8); because we have established in the Appendix that the discriminant $\left(C_{1}^{2}-4C_{0}C_{2}\right)$ is non‐positive, *f* ^−1^(*x*; *C*_0_, *C*_1_, *C*_2_) is increasing in *x*, and hence (8) provides a confidence interval where the lower bound is less than the upper bound. If both the confidence bounds are negative (which is possible), then they would be truncated to zero in practice; this means that the resulting confidence interval can include 0 and could be [0, 0], as in the exact method (Jackson, 2013; Jackson *et al.*, 2014). This truncation is also conventionally performed for the estimate ${\hat{\tau }}_{a}^{2}$. The resulting point estimate of *τ* ^2^ is then guaranteed to lie in the confidence interval and is a natural point estimate to accompany the confidence interval given by (8). Alternative conventions to setting the confidence interval to [0, 0] when both the confidence bounds are negative are to report this interval as the empty set or instead conclude that the interval is undefined. This statement would be accompanied with a conclusion like the data appear to be highly homogenous or that the interval estimation fails (Jackson *et al.,* 2014).

### 3.3 A comparison with other approaches

Because *Q*_*a*_, and hence ${\hat{\tau }}_{a}^{2}$, are typically positively skewed, a natural idea is to use a log transformation for one of these statistics. The transformation *f*(⋅) involves a log function and so is not dissimilar to this idea. However, a simple log transformation for ${\hat{\tau }}_{a}^{2}$ is not possible when this estimate is negative. Similarly, Higgins and Thompson (2002), in their Appendix A2, suggested obtaining intervals for their *H* ^2^ statistic (the ratio of Cochran's *Q* statistic and its degrees of freedom *v*) using a standard error for log(*H* ^2^) and a normal approximation on the log(*H* ^2^) scale. Because log(*H* ^2^) = log(*Q*/*v*) = log(*Q*) − log(*v*), this idea is very similar to using a log transformation on log(*Q*_*a*_). However, Higgins and Thompson provide two forms of the standard error of *H* ^2^, where the one used in practice depends on the value of *H* ^2^ (Higgins and Thompson, 2002, page 1554). By basing our intervals on the variance‐stabilising transformation of ${\hat{\tau }}_{a}^{2}$, we can use our approximation regardless of its magnitude.

Biggerstaff and Tweedie (1997) and Biggerstaff and Jackson (2008) suggest alternative approximations to the true density of Cochran's *Q* statistic. Extending these for use with *Q*_*a*_ so that approximate confidence intervals could be produced in a similar way is a possible avenue for further work. In particular, the gamma approximation to Cochran's *Q* statistic (Biggerstaff and Tweedie, 1997; Biggerstaff and Jackson, 2008) is also based on the first two moments of *Q*. However, the approach suggested here, which uses a normal approximation, is conceptually and computationally much simpler than this and other alternative approximate methods. Furthermore, the approach we propose here is motivated by variance‐stabilising transformations and so is especially well grounded in well established statistical practice.

## 4 Simulation study

In order to investigate the properties of our proposal, the simulation study performed by Jackson (2013) was reproduced using the proposed method to provide approximate 95% confidence intervals. This simulation study explores how well the approximation works for meta‐analyses (no covariates) and simulates data under the random effects model. Results using the exact method and 40 000 simulated datasets for each set of parameters were previously given by Jackson (2013), where some of these results were presented in the Supporting Information. Here, the simulation study was performed using 100 000 simulated datasets, in order to further reduce the Monte Carlo error and to ensure that all methods were applied to the same simulated datasets. All results presented here are within Monte Carlo error of those presented previously by Jackson (2013). The aim of this simulation study was to investigate how well the proposed method performs in the simulation study design presented previously.

The within‐study variances were obtained as the (0, 1, 2, …, (*n* − 1)) × 100/(*n* − 1)% quantiles of the scaled and truncated *χ* ^2^ distribution originally used by Brockwell and Gordon (2001; 2007) for producing within‐study variances. Specifically, the within‐study variances were taken as these quantiles from $0.25\times \chi_{1}^{2}$, where this random variable is further truncated to lie within the interval [0.009, 0.6]. Brockwell and Gordon (2001) state that this distribution of within‐study variances is consistent with the typical distribution of within‐study variances for log‐odds ratios in practice. Hence, we also use this distribution here, but more realistic distributions for within‐study variances, and distributions that are consistent with other outcomes, could be explored in future simulation studies. Further simulation studies could also allow for an association between the within‐study variances and the estimated effects. By using quantiles from Brockwell and Gordon's distribution, rather than random observations, we have fixed within‐study variances for each value of *n*. This facilitates the interpretation of values of *τ* ^2^ as corresponding to fixed values of *I* ^2^. Data were simulated using a true treatment effect of zero, but this choice is immaterial.

Five values of *τ* ^2^ = 0, 0.029, 0.069, 0.206, 1.302 were investigated, because they correspond to *I* ^2^ = 0, 0.30, 0.50, 0.75, 0.95 (Higgins *et al*., 2009) when *n* = 5 and so cover a wide range of possibilities. Meta‐analyses with *n* = 5, 10, 20, 40 studies were simulated, and both the standard weights $a_{i}=1/\sigma_{i}^{2}$ and the weights *a*_*i*_ = 1/*σ*_*i*_ proposed by Jackson (2013) were used. The use of *a*_*i*_ = 1/*σ*_*i*_ allows large studies to have more weight than small ones, but this weight is distributed more evenly than in the fixed‐effect weights ( $a_{i}=1/\sigma_{i}^{2}$), which is likely to be more appropriate if heterogeneity is present. Results using the *Q* profile method (Knapp *et al*, 2006; Viechtbauer, 2007) were also obtained using the *metafor* package so that readers can compare the main results to those obtained using this method.

The average length of 100 000 exact and approximate confidence intervals for each combination of *n* and *τ* ^2^ were calculated, and the coverage probabilities of the approximate 95% confidence intervals were estimated by the proportion of intervals that contain the true values. As explained by Jackson (2013), the exact method provides the correct nominal coverage probability of 0.95 if *τ* ^2^ > 0. If *τ* ^2^ = 0, however, the exact method is conservative and produces a coverage probability of 0.975 (Jackson, 2013).

The main results of the simulation study are shown in Table 1. The approximate method is consistently even more conservative than the exact method when *τ* ^2^ = 0. This can be explained because, before truncation, the normal approximation that underlies the proposed approximation can include negative values of *τ* ^2^ within the confidence interval. Hence, the true value of *τ* ^2^ = 0 is included in the approximate confidence intervals more often. This means that the approximate method can be conservative when *τ* ^2^ is small. However, the approximate method provides confidence intervals that are, on average, shorter than the exact method. Because the exact confidence intervals are bounded from below at zero, the very long exact confidence intervals must be because of the large upper bounds. This means that larger values of *τ* ^2^ are less likely to be included in the approximate, than the exact, confidence intervals. This has some unfortunate implications for the coverage probabilities of confidence intervals obtained using the approximate method when *τ* ^2^ is large and *n* is small. Together these observations make it clear that the variance‐stabilising transformation is not able to fully counteract the skewness of *Q*_*a*_ statistics across the entire range of *τ* ^2^ in small samples, but it is perhaps unrealistic to expect it to be completely successful in this respect. As noted by Jackson (2013), *a*_*i*_ = 1/*σ*_*i*_ provides shorter confidence intervals than $a_{i}=1/\sigma_{i}^{2}$ unless the extent of the between‐study heterogeneity is small. Hence, the best choice of the two possibilities explored for the *a*_*i*_ depends on the magnitude of *τ* ^2^.

**Table 1:** Results from the simulation study.

Sample size and weights	*τ* ^2^ = 0			*τ* ^2^ = 0.029			*τ* ^2^ = 0.069			*τ* ^2^ = 0.206			*τ* ^2^ = 1.302		
	*L*_E_	*L*_A_	*C*_A_	*L*_E_	*L*_A_	*C*_A_	*L*_E_	*L*_A_	*C*_A_	*L*_E_	*L*_A_	*C*_A_	*L*_E_	*L*_A_	*C*_A_
*n* = 5 $a_{i}=1/\sigma_{i}^{2}$	0.801	0.302	0.999	1.211	0.497	0.973	1.757	0.770	0.920	3.665	1.757	0.897	18.905	9.970	0.917
*n* = 5 *a*_*i*_ = 1/*σ*_*i*_	0.976	0.425	0.995	1.257	0.548	0.997	1.633	0.723	0.993	2.941	1.368	0.928	13.296	6.687	0.910
*n* = 5 (*Q* profile)	1.423	—	—	1.678	—	—	2.008	—	—	3.135	—	—	11.881	—	—
*n* = 10 $a_{i}=1/\sigma_{i}^{2}$	0.194	0.114	0.997	0.347	0.227	0.938	0.550	0.387	0.923	1.254	0.953	0.929	6.887	5.498	0.944
*n* = 10 *a*_*i*_ = 1/*σ*_*i*_	0.267	0.183	0.988	0.374	0.256	0.979	0.515	0.358	0.958	0.993	0.722	0.932	4.722	3.624	0.933
*n* = 10 (*Q* profile)	0.363	—	—	0.471	—	—	0.604	—	—	1.036	—	—	4.235	—	—
*n* = 20 $a_{i}=1/\sigma_{i}^{2}$	0.080	0.058	0.993	0.163	0.130	0.939	0.269	0.227	0.935	0.633	0.561	0.941	3.561	3.251	0.950
*n* = 20 *a*_*i*_ = 1/*σ*_*i*_	0.123	0.102	0.983	0.186	0.154	0.962	0.269	0.224	0.950	0.522	0.450	0.941	2.519	2.239	0.942
*n* = 20 (*Q* profile)	0.145	—	—	0.215	—	—	0.295	—	—	0.539	—	—	2.288	—	—
*n* = 40 $a_{i}=1/\sigma_{i}^{2}$	0.041	0.034	0.988	0.096	0.085	0.943	0.159	0.147	0.942	0.379	0.358	0.945	2.162	2.075	0.949
*n* = 40 *a*_*i*_ = 1/*σ*_*i*_	0.069	0.063	0.979	0.115	0.105	0.954	0.169	0.155	0.949	0.322	0.300	0.945	1.571	1.486	0.947
*n* = 40 (*Q* profile)	0.069	—	—	0.121	—	—	0.175	—	—	0.331	—	—	1.434	—	—

Simulated datasets (100 000) were produced for each value of *τ* ^2^. The average lengths of the exact 95% confidence intervals are denoted as *L*_E_, and the average length of the corresponding approximate 95% confidence intervals are denoted as *L*_A_. The exact method provides coverage probabilities of 0.95 for *τ* ^2^ > 0 and 0.975 for *τ* ^2^ = 0. The estimated coverage probabilities of the approximate 95% confidence intervals are denoted as *C*_A_. The average length of 95% confidence intervals using the *Q* profile method are provided for comparison.

The finding that the proposed approximate method can provide shorter confidence intervals but also that it can provide larger confidence probabilities for small *τ* ^2^ may appear curious. In order to investigate this further, in Table 2 we show the average 95% confidence interval lower bounds and the estimated probabilities that the confidence interval lower bounds are greater than the true value of *τ* ^2^ (so that the entire confidence interval lies to the right of the true value). For the exact and *Q* profile methods, the estimated probabilities that the confidence interval lower bounds are greater than than the true value of *τ* ^2^ were found to be within Monte Carlo error of 0.025, as theory dictates. The probability that the confidence intervals lie to the left of the true value of *τ* ^2^ can be obtained from the results in Tables 1 and 2 as 1 − *C*_A_ − *M*_A_. The average upper bounds can also be obtained from these results as the average lower bounds plus the average confidence interval lengths. For *τ* ^2^ = 0, we have *C*_A_ + *M*_A_ = 1, because if there is no between‐study heterogeneity, then confidence intervals either contain the true value of *τ* ^2^ = 0 or lie to the right of this value.

**Table 2:** Further results from the simulation study.

Sample size and weights	*τ* ^2^ = 0			*τ* ^2^ = 0.029			*τ* ^2^ = 0.069			*τ* ^2^ = 0.206			*τ* ^2^ = 1.302		
	Low_E_	Low_A_	*M*_A_	Low_E_	Low_A_	*M*_A_	Low_E_	Low_A_	*M*_A_	Low_E_	Low_A_	*M*_A_	Low_E_	Low_A_	*M*_A_
*n* = 5 $a_{i}=1/\sigma_{i}^{2}$	3 × 10^− 4^	3 × 10^− 6^	0.001	0.002	1 × 10^− 4^	0.006	0.007	0.001	0.008	0.036	0.010	0.001	0.319	0.148	0.001
*n* = 5 *a*_*i*_ = 1/*σ*_*i*_	9 × 10^− 4^	1 × 10^− 4^	0.005	0.002	2 × 10^− 4^	0.003	0.005	8 × 10^− 4^	0.002	0.029	0.009	0.002	0.364	0.201	0.002
*n* = 10 $a_{i}=1/\sigma_{i}^{2}$	2 × 10^− 4^	1 × 10^− 5^	0.003	0.003	6 × 10^− 4^	0.003	0.011	0.004	0.003	0.054	0.029	0.004	0.423	0.282	0.005
*n* = 10 *a*_*i*_ = 1/*σ*_*i*_	6 × 10^− 4^	2 × 10^− 4^	0.012	0.002	7 × 10^− 4^	0.008	0.006	0.003	0.006	0.048	0.029	0.005	0.514	0.395	0.007
*n* = 20 $a_{i}=1/\sigma_{i}^{2}$	1 × 10^− 4^	3 × 10^− 5^	0.007	0.004	0.002	0.006	0.017	0.010	0.007	0.076	0.057	0.008	0.546	0.448	0.009
*n* = 20 *a*_*i*_ = 1/*σ*_*i*_	4 × 10^− 4^	2 × 10^− 4^	0.017	0.002	0.001	0.013	0.010	0.006	0.011	0.074	0.059	0.009	0.660	0.583	0.011
*n* = 40 $a_{i}=1/\sigma_{i}^{2}$	1 × 10^− 4^	4 × 10^− 5^	0.012	0.006	0.004	0.010	0.025	0.020	0.012	0.100	0.088	0.013	0.684	0.624	0.014
*n* = 40 *a*_*i*_ = 1/*σ*_*i*_	3 × 10^− 4^	2 × 10^− 4^	0.021	0.003	0.002	0.017	0.016	0.013	0.015	0.102	0.093	0.014	0.796	0.750	0.015

Simulated datasets (100 000) were produced for each value of *τ* ^2^. The average lower bounds of the exact 95% confidence intervals are denoted as Low_E_, and the average lower bounds of the corresponding approximate 95% confidence intervals are denoted as Low_A_. The estimated probability that the approximate lower bound is greater than the true *τ* ^2^ is denoted by *M*_A_.

From Table 2, we can see that the approximate method consistently provides slightly, but noticeably, smaller lower confidence interval bounds. This means that the shorter average confidence interval lengths from the proposed approximate method is entirely due to it providing much smaller average values of the upper confidence interval bounds. This provides more evidence that the variance‐stabilising transformation is not able to fully counteract the skewness of *Q*_*a*_ statistics across the entire range of *τ* ^2^. The larger than 95% coverage probabilities of the approximate method when *τ* ^2^ is small can be explained by the finding that the approximate method provides, on average, slightly smaller confidence interval lower bounds than the exact method. The exact method is conservative when *τ* ^2^ = 0, which partly explains why the approximate method is also conservative when the data are homogenous. The proposed approximate method generally departs substantially from providing equal tailed confidence intervals and in small samples is, at times, almost one‐tailed. This observation suggests that the results using the proposed method are not necessarily directly comparable to those obtained using the exact method; this is because we have not investigated the use of the exact method with unequal tailed confidence intervals, and this may form the subject of future work.

One interpretation of the results in Tables 1 and 2 is that the approximation only gives similar results (especially with regard to the average length of the confidence intervals) to the exact method when *n* is really quite large; even for *n* = 40, we can see from Tables 1 and 2 that the results using approximate method do not match the exact method very closely. A more positive interpretation however is that the estimated coverage probabilities of the approximate 95% confidence intervals in Table 1 do not drop below around 90%. Hence, the actual coverage probability of the approximate method is likely to be considered satisfactory in practice.

It should be recognised that the simulation study completely adheres to the random‐effects model. The simulation study therefore investigates the properties of the proposed method in an idealised setting where all the assumptions are true. Further simulation studies are needed to evaluate all the various methods for calculating confidence intervals for *τ* ^2^ because the conclusions from our simulation study may not generalise to other settings. Our aim here was to see how the proposed method performs in the setting previously investigated (Jackson, 2013). In particular, further simulation studies could investigate alternative distributions of within‐study variances that are intended to correspond to other measures of treatment effect and further could explore the properties of all the various proposals in situations where the random effects model only provides an approximation. We also leave an investigation of how well the proposed method performs in practice for meta‐regression as an avenue for future work.

## 5 Application to examples

In order to further examine the use of the approximate method, we applied both this and the exact method to nine real datasets. Eight of these represent the type of dataset we encounter quite often, where there are quite small numbers of studies. We also include one unusually very large dataset to examine a situation where we would expect our approximation to work well. Hence, if the proposed method cannot perform well here, then it is unlikely to perform well anywhere. Now that we have real data, the random‐effects model only provides an approximation, and hence, the ‘exact’ confidence intervals are also in reality only approximate.

Four of the examples (Aspirin, Glycerol, Duiretic and Sclerotherapy) are somewhat ‘historic’ examples, where we took the study specific estimates and within‐study variances from Biggerstaff and Jackson (2008) and Jackson (2013). We provide results for these examples so that they be compared with those given in previous papers using the exact method. Four of the other examples relate to cancer (Cervix3, Nsclc4, Nsclc1 and Cervix1) and are from Bowden *et al.* (2011). The last, very large, example with 111 studies (smoking) is from Baker and Jackson (2013).

The results in Table 3 reinforce the conclusion from Table 1 that the approximate 95% confidence interval is only in good agreement with the exact confidence interval when the sample size is large enough. The approximate 95% confidence intervals are much shorter than the exact ones for the four examples where *n* < 10. The approximate confidence intervals are in better agreement with exact ones for the four examples where 10 ≤ *n* ≤ 20 (but are still considerably shorter). As we would hope and anticipate, the approximate and exact confidence intervals are in very good agreement for the final very large dataset. Furthermore, no approximate confidence bound in this empirical investigation is greater than the corresponding exact confidence bound, which also supports the conclusions from the simulation study. To summarise, both the simulation study and this empirical investigation reassure us that the proposed method performs satisfactorily. However, meta‐analyses with fewer than 10 studies are the general rule in many application areas. The case for using the approximate method proposed here, instead of the previously developed exact method, is weakest in such situations.

**Table 3:** Results for the nine examples using the exact and proposed approximate methods.

Data set	*n*	*I* ^2^ (%)	$a_{i}=1/\sigma_{i}^{2}$			*a*_*i*_ = 1/*σ*_*i*_		
			${\hat{\tau }}_{a}^{2}$	Exact CI	Approx CI	${\hat{\tau }}_{a}^{2}$	Exact CI	Approx CI
Cervix3	5	56	0.087	(0, 1.372)	(0, 0.633)	0.104	(0, 1.464)	(0, 0.663)
Aspirin	6	49	0.027	(0, 0.339)	(0, 0.181)	0.012	(0, 0.235)	(0, 0.119)
Glycerol	9	23	0.079	(0, 1.124)	(0, 0.688)	0.011	(0, 1.012)	(0, 0.663)
Diuretic	9	71	0.230	(0.047, 1.431)	(0.014, 1.056)	0.329	(0.074, 1.678)	(0.036, 1.179)
Nsclc4	11	75	0.132	(0.040, 0.559)	(0.026, 0.419)	0.170	(0.055, 0.651)	(0.039, 0.493)
Nsclc1	17	45	0.024	(0.000, 0.118)	(0, 0.093)	0.035	(0, 0.147)	(0, 0.118)
Cervix1	18	62	0.112	(0.032, 0.370)	(0.021, 0.308)	0.144	(0.043, 0.438)	(0.031, 0.368)
Sclerotherapy	19	56	0.302	(0.071, 1.023)	(0.040, 0.854)	0.231	(0, 0.867)	(0, 0.724)
Smoking	111	26	0.038	(0.006, 0.092)	(0.004, 0.087)	0.043	(0, 0.110)	(0, 0.105)

${\hat{\tau }}_{a}^{2}$ denotes the estimated between‐study variance. Exact CI and Approx CI denote 95% confidence intervals for *τ* ^2^ using the exact and approximate methods. A lower bound of 0 means that this was truncated to zero. *I* ^2^ denotes the conventional heterogeneity statistic. The estimated effects for the Cervix3, Nsclc4, Nsclc1 and Cervix1 datasets are log hazard ratios. For the other five datasets, the estimated effects are log odds ratios.

The R code that produces the results using the proposed method in Table 3 is available in the Supporting Information. The R code in the Supporting Information also contains the data used in analysis for all nine examples and forest plots that illustrate these data.

## 6 Conclusions

The proposed approximate approach for constructing confidence intervals for the between‐study variance is conceptually and computationally simple. The simulation study suggests that the proposed approximate confidence interval does not deviate far from the intervals' nominal level of 95% over the wide range of possibilities explored. However, the approximate method appears to result in shorter confidence intervals than the corresponding exact method, so it should not be assumed that the approximate interval necessarily agrees very well with the exact method unless the number of studies is really quite large. Narrower confidence intervals that are able to retain the nominal coverage probability, and the same allocation of non‐coverage between the two tails, are preferable to longer confidence intervals; our approximate method results in narrower confidence intervals but does not quite retain the nominal coverage probability. The exact method is quite computationally intensive in very large sample sizes, and so the proposed method is especially advantageous in meta‐analyses where there are very many studies.

The proposed approach means that interval estimation for the between‐study variance can now be as straightforward as the point estimation. This has hitherto not been the case, so we feel that our proposed approach will be attractive to many meta‐analysts. The methodology provides an approach that is accessible to applied researchers who may not have access to Farebrother's algorithm or iterative methods. Furthermore, the bounds of the proposed approximate confidence interval, like the point estimate, are functions of ${\hat{\tau }}_{a}^{2}$ and hence are also functions of *Q*_*a*_. The approximate approach therefore makes the calculation of confidence intervals for the between‐study variance much more transparent than other methods.

We presented our methods in the context of the random‐effects meta‐regression model. Meta‐regression provides a framework for some types of network meta‐analysis (White *et al*., 2012), and so our method is also potentially useful in the network meta‐analysis setting.

Although the method for calculating confidence intervals developed here is only approximate, the random effects model only provides an approximation to real data such as that in Section 5. The value of an exact method is diminished when the approximations made in the modelling process are quite crude. Hence, there will be many situations where the proposed approximation is likely to be considered to be adequate. The method developed here in no way diminishes the exact method, but we feel that, at the very least, the proposed approximate method adds another useful technique to the ever expanding meta‐analysis armoury that we now have at our disposal.

## Supporting information



Supporting info item

Supporting info item

Supporting info item

## Appendix 1

We will show that the surds in (7) present no difficulties. For this we require that *g*(*x*) = *C*_0_ + *C*_1_ *x* + *C*_2_ *x* ^2^ ≥ 0 for *x* ≥ − tr(**B**Δ)/tr(**B**), but it is easier to prove the stronger condition that *g*(*x*) is nonnegative for all *x*. The discriminant of the quadratic equation *g*(*x*) is $C_{1}^{2}-4C_{0}C_{2}$, and if this discriminant is non‐positive, then *g*(*x*) has either no real roots or a single root of zero. Furthermore, the leading coefficient, *C*_2_, of this quadratic is positive because tr(**B** ^2^) ≥ 0. This is because **B** is positive semi‐definite and hence so is **B** ^2^ (a consequence of Zhang, theorem 7.5.2), and so tr(**B** ^2^) ≥ 0. Hence, if the discriminant $C_{1}^{2}-4C_{0}C_{2}$ is non‐positive, then *g*(*x*) ≥ 0 for all *x*. This condition requires

(9) $${\left(tr\left(B\Delta B\right)\right)}^{2}\leq tr\left(B\Delta B\Delta \right)tr\left(B^{2}\right)$$

The result on page 213 of Zhang can be used to establish (9). This result states that if **C** and **D** are positive semi‐definite matrices of the same size, then tr(**C** ^1/2^ **D** ^1/2^) ≤ (tr(**C**))^1/2^(tr(**D**))^1/2^, where **C** ^1/2^ and **D** ^1/2^ are the square roots of **C** and **D** (Zhang, 2010). This is a special case of the Cauchy–Schwarz inequality. Using this result with **C** = (**B** ^1/2^Δ**B** ^1/2^)^2^ and **D** = **B** ^2^ results in (9), so that *g*(*x*) ≥ 0.
