# Supplementary material for: Approximate confidence intervals for moment‐based estimators of the between‐study variance in random effects meta‐analysis
Source: Res Synth Methods. 2015 Aug 19;6(4):372–82. doi: 10.1002/jrsm.1162 (PMC4839498; doi:10.1002/jrsm.1162)

**Forest plots for the nine example datasets**

1. **Cervix3. Outcome: Log hazard ratio**


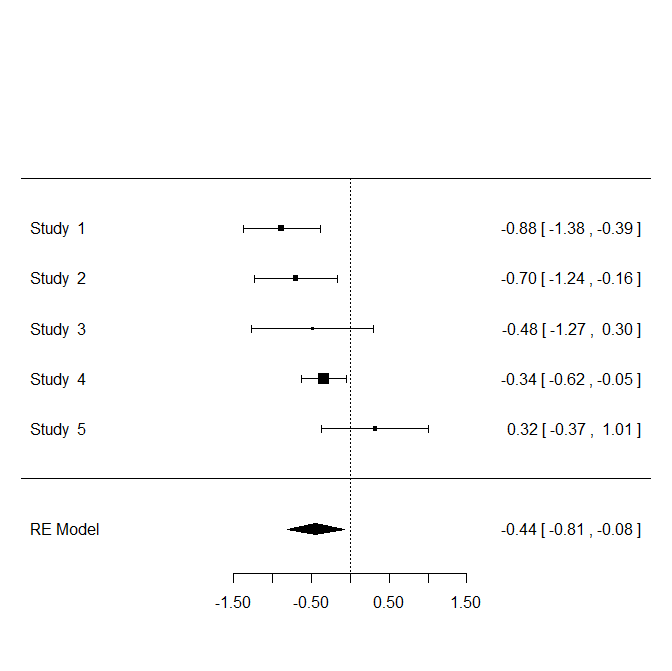


1. **Aspirin. Outcome: Log odds ratio**


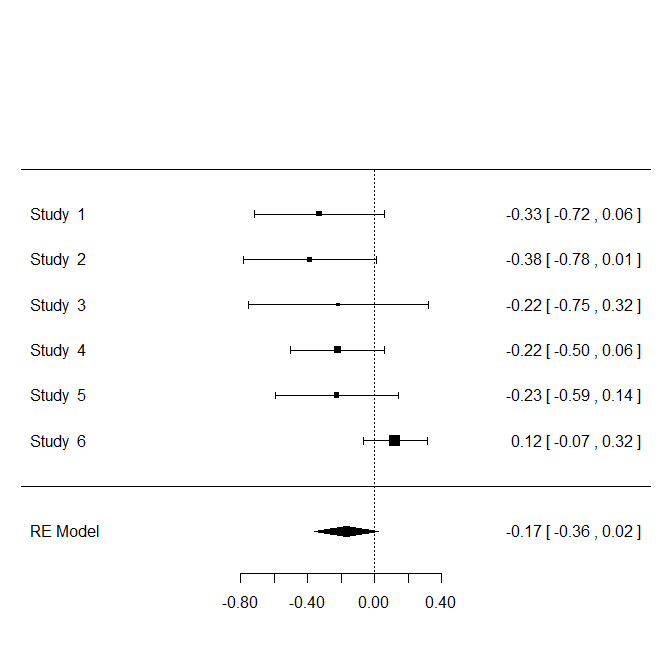


1. **Glycerol. Outcome: Log odds ratio**


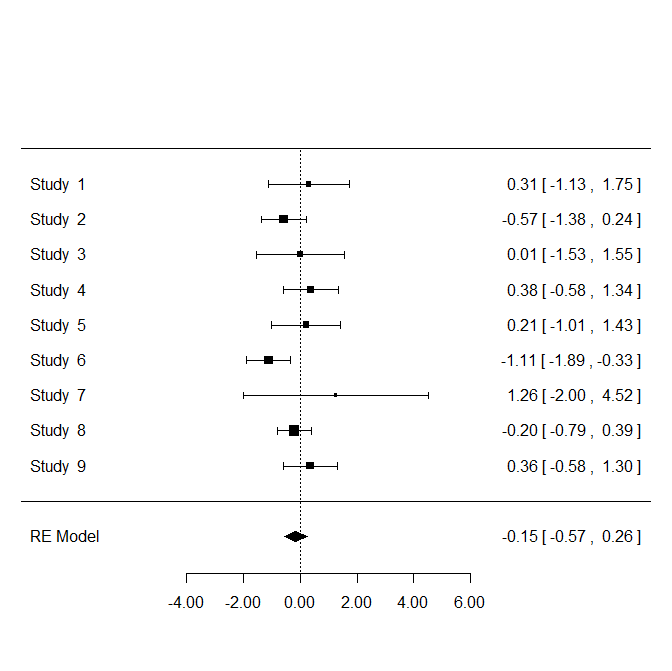


1. **Duietic. Outcome: Log odds ratio**
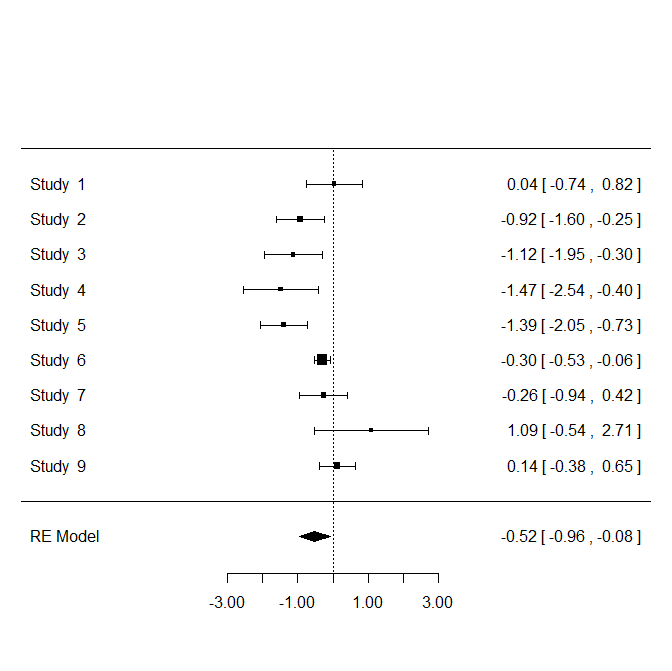

2. **Nsclc4. Outcome: Log hazard ratio**.
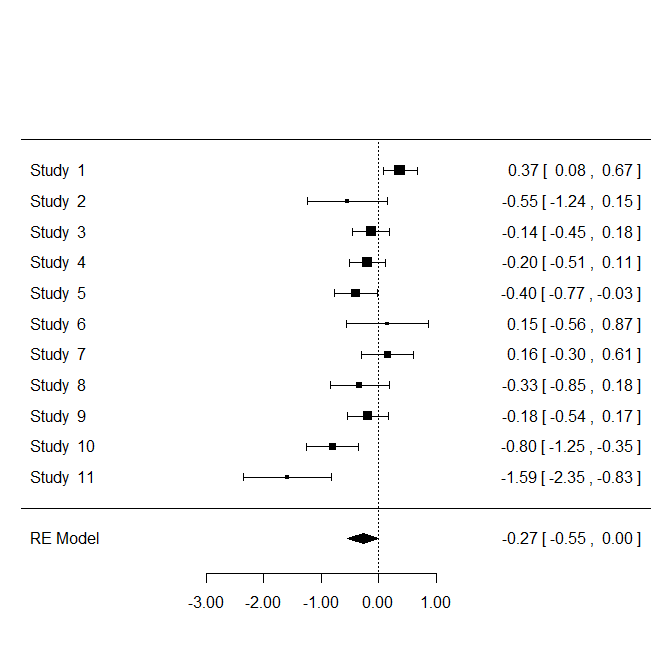


**6. Nsclc1. Outcome Log hazard ratio**
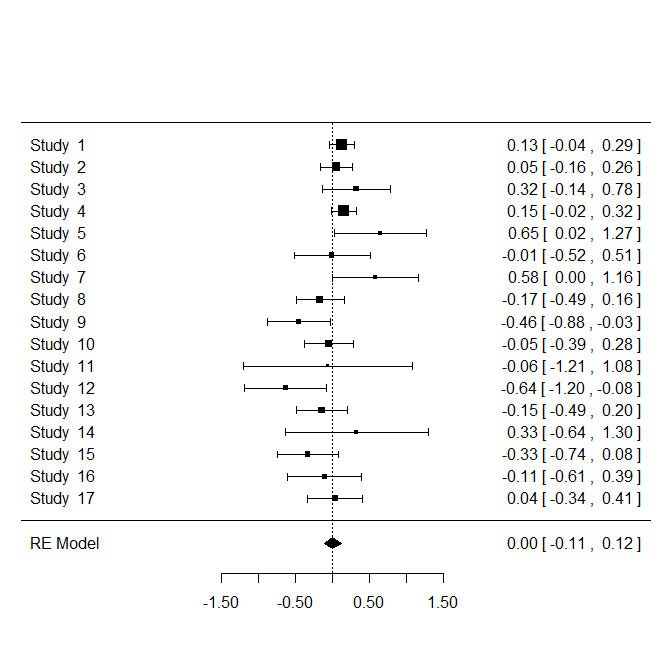


**7. Cervix1. Outcome: log odds ratio**
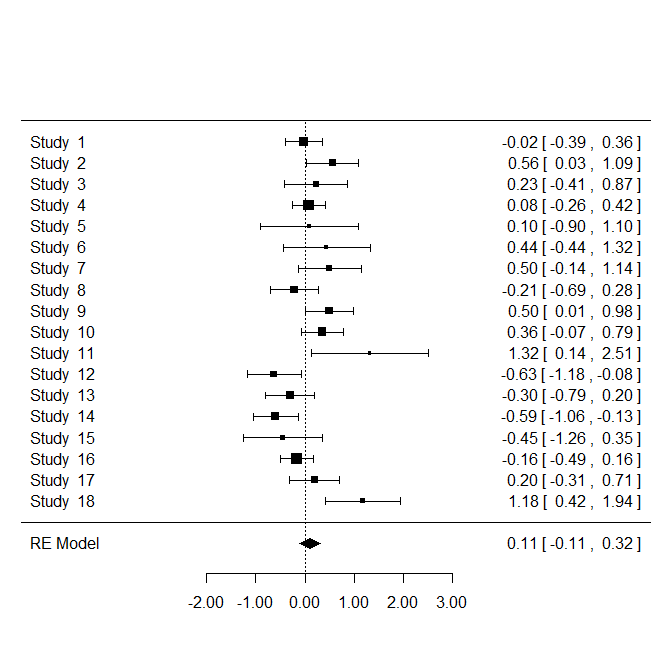


**8. Sclerotherapy. Outcome: log odds ratio**


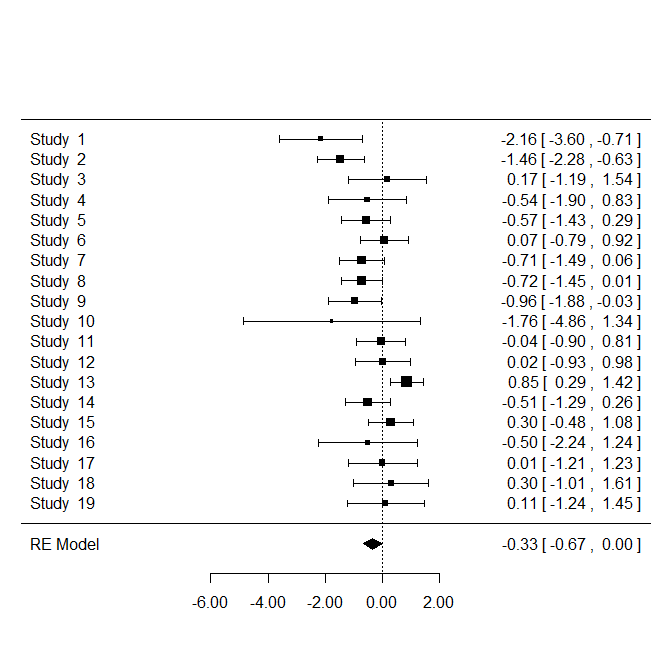


**9. Smoking. Outcome: log odds ratio**
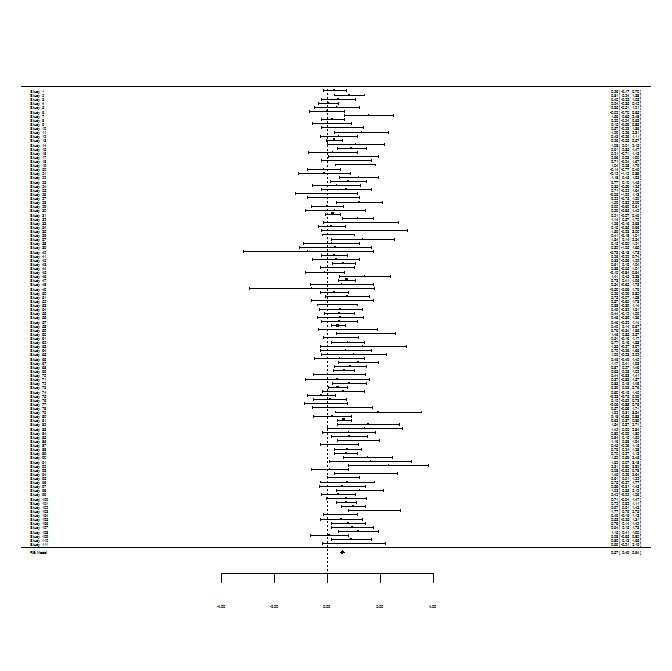

Supplement: Supplementary file 1 — Supporting info item [file JRSM-6-372-s001.docx]
