# Supplementary material for: Approximate confidence intervals for moment‐based estimators of the between‐study variance in random effects meta‐analysis
Source: Res Synth Methods. 2015 Aug 19;6(4):372–82. doi: 10.1002/jrsm.1162 (PMC4839498; doi:10.1002/jrsm.1162)
Supplement: Supplementary file 3 — Supporting info item [file JRSM-6-372-s003.doc]

**R CODE**

This R code produces the results, using the proposed methodology, in table 3 of the paper; the datasets are also provided below. Code for implementing the exact method was provided previously by Jackson (2013).

The function “inference_approx” is called to produce the results, where inference_approx is a function of five arguments. These five arguments are:

**y** and **v** – the outcomes and the within-study variances.

**power** and **con** – power is denoted by p in Jackson (2013) and con is the constant (denoted by *x* in the Jackson 2013) added to the within-study variances when computing the weights. The code therefore allows weights of the form

$$a_{i}=(\sigma_{i}^{2}+con)^{power}$$

So power = 1 and con = 0, and power = 0.5 and con = 0, produce the results using the proposed method in the paper.

**coverage** is the coverage probability of the interval, so coverage=0.95 is conventional.

The function returns three objects: **tau2** (the estimate of the between study-variance, as proposed by DerSimonian and Kacker, 2007), **CI** (the confidence interval) and **width** (which is just the upper bound of the confidence interval minus the lower bound).

Copy and paste the code below into R to produce the results in table 3 using the proposed methodology.

inference=function (y, v, power, con, cover=0.95)

{

n=length(y)

alpha=(1-cover)/2

Z=-qnorm(alpha)

a = (1/(v+con))^power

A = diag(a)

a=matrix(a, ncol=1)

B = A - a%*%t(a)/sum(a)

Y = matrix(y, ncol = 1)

QE = t(Y) %*% B %*% Y

Delta=diag(v)

dof=sum(diag(B%*%Delta))

constant=sum(diag(B))

C_2=2*sum(diag(B%*%B))/constant^2

C_1=4*sum(diag(B%*%Delta%*%B))/constant^2

C_0=2*sum(diag(B%*%Delta%*%B%*%Delta))/constant^2

tau2=max((QE-dof)/constant,0)

Ans=(QE-dof)/constant

res=f(Ans, A=C_2, B=C_1, C=C_0)

lower=max(f_inverse(res-Z, A=C_2, B=C_1, C=C_0),0); upper=max(f_inverse(res+Z, A=C_2, B=C_1, C=C_0),0)

return(list(tau2=tau2, CI=c(lower, upper), width=upper-lower))

}

f=function (x, A, B, C)

{

part1=(A*(A*x^2+B*x+C))^0.5

(1/A^0.5)*log(2*A*x+B+2*part1)

}

f_inverse=function (x, A, B, C)

{

(exp(A^0.5*x)-2*B+(B^2-4*A*C)*exp(-A^0.5*x))/(4*A)

}

Y1=c(-0.882428115015974, -0.699400299850075, -0.483091787439614,

-0.335836909871245, 0.321428571428571)

V1=c(0.0638977635782748, 0.0749625187406297, 0.161030595813205,

0.0214592274678112, 0.123152709359606)

Y2=c(-0.328901159556238, -0.384545745884422, -0.215762453588579,

-0.218181972954969, -0.225467203812011, 0.124636302494911)

V2=c(0.0388956529883766, 0.0411672601083638, 0.0753454212023551,

0.0204896028636616, 0.0351996442150119, 0.00961673238271602)

Y3=c(0.31, -0.57, 0.01, 0.38, 0.21, -1.11, 1.26, -0.20, 0.36)

V3=c(0.54, 0.17, 0.62, 0.24, 0.39, 0.16, 2.77, 0.09, 0.23)

Y4=c(0.0418471099355005, -0.923670839171778, -1.1221427860783, -1.47330573810952, -1.39102453549529, -0.296889451161883, -0.261549932174933, 1.0887599922251, 0.135305386348586)

V4=c(0.159600872715627, 0.117736838325074, 0.178017718715393, 0.298926767676768, 0.11428507325059, 0.01463367728029, 0.120687453528594, 0.686371578334304, 0.0678772840063163)

Y5= c(0.374429223744292, -0.548185231539424, -0.135526315789474,

-0.201802704056084, -0.39943342776204, 0.15364238410596, 0.156517094017094,

-0.332415691672402, -0.184814216478191, -0.798082045817794,

-1.59152798789713)

V5= c(0.0228310502283105, 0.125156445556946, 0.0263157894736842,

0.0250375563345018, 0.0354107648725213, 0.132450331125828, 0.0534188034188034, 0.0688231245698555, 0.0323101777059774, 0.053276505061268, 0.151285930408472)

Y6= c(0.127128104939994, 0.0523255813953488, 0.322259136212625, 0.150003635570421, 0.646998982706002, -0.00621976503109882, 0.580105633802817, -0.168881506090808, -0.455560725919032, -0.0529085062847121, -0.0648464163822526, -0.638001638001638, -0.145985401459854, 0.326781326781327, -0.333914559721011, -0.108409986859396, 0.0357925493060628)

V6= c(0.00697739324588334, 0.0116279069767442, 0.0553709856035437,

0.00727114084199811, 0.101729399796541, 0.0691085003455425, 0.0880281690140845, 0.0276854928017719, 0.0465332712889716, 0.0292312189418299, 0.341296928327645, 0.0819000819000819, 0.0304136253041363, 0.245700245700246, 0.04359197907585, 0.0657030223390276, 0.0365230094959825)

Y7= c(-0.0169920462762111, 0.560117302052786, 0.230605738575983,

0.0802716887928373, 0.0963541666666667, 0.439918533604888, 0.501607717041801, -0.207926829268293, 0.49540159411404, 0.358417752050169, 1.32234432234432, -0.628053585500394, -0.296272493573265, -0.593068753493572, -0.451178451178451, -0.163504464285714, 0.19946452476573, 1.18373493975904)

V7= c(0.0361532899493854, 0.0733137829912023, 0.106269925611052,

0.0308737264587836, 0.260416666666667, 0.203665987780041, 0.107181136120043, 0.0609756097560976, 0.0613120784794605, 0.0482392667631452, 0.366300366300366, 0.0788022064617809, 0.06426735218509, 0.0558971492453885, 0.168350168350168,

0.0279017857142857, 0.0669344042838019, 0.150602409638554)

Y8=c(-2.156, -1.455, 0.174, -0.535, -0.568, 0.067, -0.712, -0.717,

-0.956, -1.76, -0.042, 0.024, 0.852, -0.511, 0.296, -0.504, 0.011, 0.303, 0.108)

V8=c(0.543, 0.177, 0.483, 0.488, 0.193, 0.189, 0.157, 0.139, 0.223,

2.501, 0.191, 0.238, 0.083, 0.156, 0.158, 0.789, 0.385, 0.448,

0.472)

Y9= c(0.262927645, 0.812116746, 0.402528248, 0.038163842, 0.348068128,

-0.023530497, 1.563075738, 0.198528838, 0.148152661, 0.565807758,

1.287448526, 0.427444015, 0.260572595, 1.081444485, 0.910928789,

0.210522992, 0.964743115, 0.710388987, 1.037552032, -0.140727659,

-0.120249128, 1.181993898, 0.774727168, 0.345302316, 0.70774598,

-0.046520016, 0.223143551, 1.202119236, 0.00291971, 0.287682072,

0.211013868, 1.144913749, 1.259139186, 0.154977406, 1.386294361,

0.412809082, 1.341173926, 0.15295314, 0.296346955, -0.727048732,

0.259152374, 0.331357136, 0.61167215, 0.378653851, -0.102675253,

1.413472682, 0.733199648, 0.538996501, -0.587786665, 0.257829109,

0.75324172, 0.565313809, 0.378653851, 0.493814278, 0.439992825,

0.481778787, 0.458953793, 0.403935967, 0.762140052, 1.460402333,

0.507281615, 0.769079235, 1.349926717, 0.698877855, 0.997968763,

0.456672266, 1.170071253, 0.865547442, 0.625663149, 0.442948201,

0.371063681, 0.832115087, 0.392557225, 0.604481543, -0.226518512,

0.101498191, -0.058956167, 0.574087821, 1.922787732, 0.179340929,

0.63104037, 1.540445041, 1.421385681, 0.797730997, 0.83521062,

1.161679139, 0.447646722, 0.758756596, 0.701742574, 1.517757501,

1.62302934, 2.309015983, 0.082238098, 1.448507546, 0.611941607,

0.752570601, 0.561087459, 1.230787749, 0.420877387, 0.712516207,

0.7161504, 0.971825299, 1.772711274, 0.48784899, 0.527632742,

0.77908961, 0.941122708, 1.154124999, 0.077386664, 0.894886823,

0.987749696)

V9= c(0.048739446, 0.083764984, 0.109622793, 0.038257029, 0.192948668,

0.118770764, 0.218487825, 0.049504432, 0.140817892, 0.163848631,

0.273147441, 0.122883781, 0.025541219, 0.299198674, 0.08056351,

0.221725114, 0.229331992, 0.237495355, 0.147670667, 0.104227799,

0.259906951, 0.147380156, 0.11999616, 0.212630575, 0.225593531,

0.360822511, 0.246978022, 0.204751598, 0.095085598, 0.336309524,

0.02078385, 0.084671831, 0.523399949, 0.066628412, 0.677631579,

0.091642197, 0.375746606, 0.290092041, 0.474422934, 1.567816092,

0.060649441, 0.20585611, 0.047178101, 0.104465624, 0.143069523,

0.242928075, 0.027479225, 0.367063492, 1.466666667, 0.077847232,

0.178228865, 0.353409091, 0.152605245, 0.173144449, 0.08164531,

0.199365786, 0.121283129, 0.018510103, 0.316190476, 0.319368132,

0.114706468, 0.098117824, 0.681783825, 0.240831965, 0.390884439,

0.232566598, 0.152129817, 0.092546517, 0.041329234, 0.244495541,

0.375144928, 0.11032955, 0.034595525, 0.163945749, 0.070749341,

0.101623916, 0.175649782, 0.336001642, 0.677426901, 0.129587027,

0.017936649, 0.356746032, 0.526546251, 0.259701168, 0.121222223,

0.15778827, 0.129710145, 0.06962639, 0.047781158, 0.223844173,

0.630983221, 0.591449545, 0.126970203, 0.370677821, 0.095193532,

0.269819466, 0.197890978, 0.203904456, 0.10583386, 0.148166272,

0.039990374, 0.055614691, 0.250306402, 0.10839599, 0.159256845,

0.105480141, 0.161549079, 0.145870331, 0.135925654, 0.153051156,

0.373839932)

inference(Y1, V1, 1, 0, 0.95)

inference(Y1, V1, 0.5, 0, 0.95)

inference(Y2, V2, 1, 0, 0.95)

inference(Y2, V2, 0.5, 0, 0.95)

inference(Y3, V3, 1, 0, 0.95)

inference(Y3, V3, 0.5, 0, 0.95)

inference(Y4, V4, 1, 0, 0.95)

inference(Y4, V4, 0.5, 0, 0.95)

inference(Y5, V5, 1, 0, 0.95)

inference(Y5, V5, 0.5, 0, 0.95)

inference(Y6, V6, 1, 0, 0.95)

inference(Y6, V6, 0.5, 0, 0.95)

inference(Y7, V7, 1, 0, 0.95)

inference(Y7, V7, 0.5, 0, 0.95)

inference(Y8, V8, 1, 0, 0.95)

inference(Y8, V8, 0.5, 0, 0.95)

inference(Y9, V9, 1, 0, 0.95)

inference(Y9, V9, 0.5, 0, 0.95)
